# An *ab initio *and AIM investigation into the hydration of 2-thioxanthine

**DOI:** 10.1186/1752-153X-4-6

**Published:** 2010-03-23

**Authors:** Xiu-Xiang Yuan, Yan-Fang Wang, Xin Wang, Wenbo Chen, John S Fossey, Ning-Bew Wong

**Affiliations:** 1 Faculty of Chemistry, Sichuan University, Chengdu, 610064, PR China; 2 School of Chemistry, University of Birmingham, Edgbaston, Birmingham, B15 2TT, UK; 3 Department of Biology and Chemistry, City University of Hong Kong, Kowloon, Hong Kong

## Abstract

**Background:**

Hydration is a universal phenomenon in nature. The interactions between biomolecules and water of hydration play a pivotal role in molecular biology. 2-Thioxanthine (2TX), a thio-modified nucleic acid base, is of significant interest as a DNA inhibitor yet its interactions with hydration water have not been investigated either computationally or experimentally. Here in, we reported an *ab initio *study of the hydration of 2TX, revealing water can form seven hydrated complexes.

**Results:**

Hydrogen-bond (H-bond) interactions in 1:1 complexes of 2TX with water are studied at the MP2/6-311G(d, p) and B3LYP/6-311G(d, p) levels. Seven 2TX^...^H_2_O hydrogen bonded complexes have been theoretically identified and reported for the first time. The proton affinities (PAs) of the O, S, and N atoms and deprotonantion enthalpies (DPEs) of different N-H bonds in 2TX are calculated, factors surrounding why the seven complexes have different hydrogen bond energies are discussed. The theoretical infrared and NMR spectra of hydrated 2TX complexes are reported to probe the characteristics of the proposed H-bonds. An improper blue-shifting H-bond with a shortened C-H bond was found in one case. NBO and AIM analysis were carried out to explain the formation of improper blue-shifting H-bonds, and the H-bonding characteristics are discussed.

**Conclusion:**

2TX can interact with water by five different H-bonding regimes, N-H^...^O, O-H^...^N, O-H^...^O, O-H^...^S and C-H^...^O, all of which are medium strength hydrogen bonds. The most stable H-bond complex has a closed structure with two hydrogen bonds (N(7)-H^...^O and O-H^...^O), whereas the least stable one has an open structure with one H-bond. The interaction energies of the studied complexes are correlated to the PA and DPE involved in H-bond formation. After formation of H-bonds, the calculated IR and NMR spectra of the 2TX-water complexes change greatly, which serves to identify the hydration of 2TX.

## Background

Hydration is a universal phenomenon in nature, many biological processes occur in aqueous media. The structure, dynamics and stability of biological macromolecules are influenced by their interactions with hydration water [1-5]. Thus, hydrogen bonds (H-bonds) between biomolecules and water play a vital role in molecular biology. Many efforts have been made to study H-bond interactions between water and nucleic acid bases, both experimentally [6-12] and theoretically [13-31]. Kong *et al*. [6] have used resonantly enhanced multiphoton ionization (REMPI) and laser-induced fluorescence (LIF) spectroscopy to study a thymine-water complex. The results indicated that hydration water can stabilize the base. Similar results were obtained for microhydrated uracil [7]. De Vries investigated the hydration of guanine base pairs and found a single water molecule suffices to stabilise the base pair structure [8]. Adamowiz and Maes reported a combined experimental and theoretical study of hydrogen-bond interactions of adenine and hypoxanthine with water [11,12]. In theoretical approaches [13-19], *ab initio *and density functional theory (DFT) calculations have been carried out to study H-bonds resulting from 1:1 complexes formed between water and uracil [14-16], cytosine [17], thymine [18-20], guanine [21-23] and adenine [11,23]. Kim and Schafer [24] investigated the microsolvation effects on the stabilities of uracil and its anion. Hobza and co-workers reported serial theoretical works on the tautomers of cytosine [25], guanine [26], adenine [27], uracil and thymine [28] in the gas phase and a microhydrated environment. Experimental and theoretical investigations on the hydration of nucleic acid bases have been reviewed by Hobza [13]. Schafer and co-workers [29-31] highlighted their theoretical explorations of the molecular mechanisms of DNA damage using quantum mechanical models. They studied electron attachment to DNA subunit anions or base pairs and found the effect of water-hydration in stabilizing the radical anions of the DNA component is crucial [31].

**Scheme 1:**
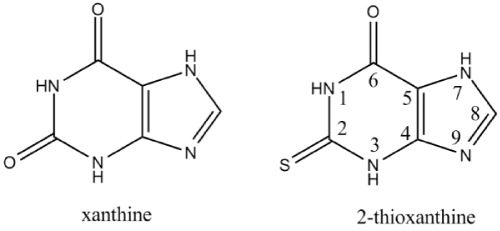
Chemical structures of xanthine and 2-thioxanthine.

Xanthines are a group clinically significant alkaloids that are commonly used as mild stimulants [32] and bronchodilator drugs to treat asthma [33,34]. 2-Thioxanthine (2TX) is a thio-modified xanthine derivative (see scheme 1).

Sulfur-substituted nucleic acid bases have been found to be clinically useful drugs [35-39], as such, 2-thioxanthine evokes intensive interest [40-48]. An earlier ^1^H NMR spectroscopic experimental study of 2TX by Twanmoh *et al*. [41] revealed that the predominant tautomer of this compound (in DMSO) is a structure in its oxothoine form with the imidazole proton on N(7), which was also corroborated by the subsequent theoretical calculations [42,43]. Spectrophotometric titration experiment by Kierdaszuk *et al*. demonstrated the p*K*_a _of 2TX is 5.9 [44]. Shugar *et al*. [45] reviewed the acid/base properties of 2TX. Tudek and co-workers [46] studied the inhibition of formamidopyrimidine-DNA glycosylases DNA base analogs and found 2TX to be the most efficient inhibitor of the seventeen tested compounds [46]. Investigations of xanthine oxidase-catalyzed reactions [47] and deactivation of xanthine permease in the presence of high affinity xanthine analogues [48] also featured 2TX.

It is noteworthy then that 2TX is of significant interest as a DNA inhibitor [45-48], and that H-bonds between 2TX and water are important. However, to the best of our knowledge, the interactions between the water of hydration and 2TX have not been investigated either computationally or experimentally.

Herein we describe *ab initio *calculations of the intermolecular interactions in 1:1 complexes of 2-thioxanthine and water, H-bond interactions in the obtained theoretical complexes are investigated and discussed. The infrared and NMR spectra are calculated to facilitate analysis of the H-bonding interactions. Their bonding characteristics are also analysed by Natural Bond Orbital (NBO) [49] and Atoms In Molecules (AIM) theory [50].

## Experimental

### Computational details

Second-order Møller-Plesset perturbation theory (MP2)[51] and density functional theory (DFT) [52] were applied to optimise the structures of the parent monomer and possible hydrated complexes and to predict the harmonic vibrational frequencies. Becke's three-parameter nonlocal exchange function and the Lee, Yang and Parr nonlocal correlation functional (B3LYP) [53,54] were employed in the DFT calculations. A moderate basis set, 6-311G(d, p), was used for optimization and frequency calculations, followed by single-point calculations with a larger 6-311++G(2df,2p) basis set to obtain more accurate energetics. The interaction energies have been calculated and the basis set superposition error (BSSE) was eliminated by the standard counterpoise (CP) correction method of Boys and Bernardi[55].

The proton affinities (PAs) and deprotonantion enthalpies (DPEs) relate to acidity and basicity of the sites involved in H-bond formation of hydrated nucleic acid bases [17]. We computed the PA and DPE to discuss why the studied complexes have different interaction energies. The PA and DPE can be defined as the negative enthalpy change of the gas-phase protonation reaction B + H^+ ^→ BH^+ ^and enthalpy change of the gas-phase deprotonation reaction AH → A^- ^+ H^+^, respectively, where the A and B represent the acid and base, respectively. The temperature-dependent enthalpy corrections were calculated at 298K and 1 atmosphere pressure.

The NMR chemical shifts for 2TX and its monohydrated complexes were calculated with the "gauge-including atomic orbital" (GIAO) method [56,57] at the MP2/6-311G(d, p) level. The chemical shift is a measure of difference in shielding (^1^H) with respect to the standard reference compound e.g. tetramethylsilane, Si(CH_3_)_4_.

NBO analysis [49] was carried out to further understand the interactions. The bonding characteristics of the different hydrogen bonded complexes were analyzed using the AIM theory of Bader [50]. Additionally the theory based on a topological analysis of the electron charge density and its Laplacian has proved invaluable for investigating the properties of H-bonding systems [58,59]. The MP2-optimized structures were used for the AIM analysis with the MP2 wave functions as input.

With the exception of the AIM analysis which was carried out with the AIM2000 program [60,61], all the calculations were performed with the Gaussian 03 package [62].

## Results and Discussion

### Structures and Interaction Energies of the 2TX^...^H_2_O complexes

Hydrogen bonds are commonly of the format X-H^...^Y, where the H atom is bound to proton donor X, and proton acceptor Y (which has a lone pair of electrons), X and Y are both electronegative atoms. The four hydrogen atoms of 2TX are composed of three N-H moieties and one C-H, which are potential proton donors for the potential acceptor, oxygen, in water. Additionally there are nitrogen, oxygen and sulfur atoms, which could serve as proton acceptors to water's H-donor potential. Thus, water could approach several different sites of 2TX to form various H-bonded complexes.

**Figure 1:**
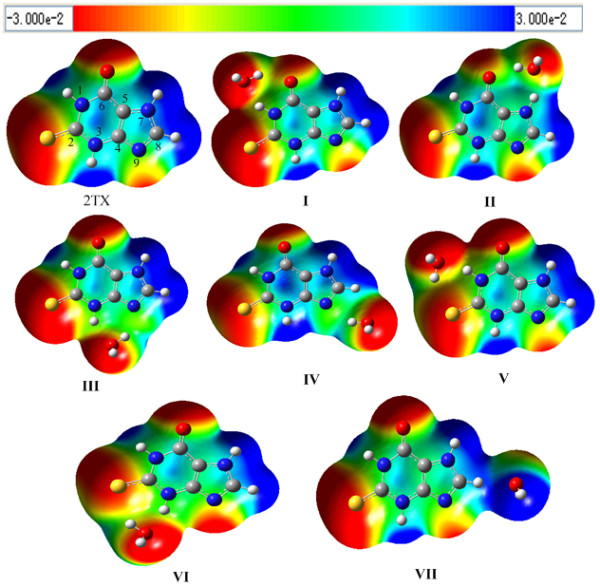
**MP2/6-311G(d, p) calculated 3D molecular electrostatic potential contour map of 2TX and its monohydrated complexes**. Red: Strong negative electrostatic potential (EP); Blue: Strong positive EP; Green: Moderately positive EP.

To better understand water's interaction with 2TX, we drew the three-dimensional (3D) molecular electrostatic potential (MEP) contour map of 2TX and its hydrated complexes, Figure 1. It can been seen that a negative electrostatic potential area exists mainly surrounding the hetero atoms O, S and N while a positive potential is concentrated in the vicinity of the hydrogen atoms. Around much of the periphery of 2TX water can approach to form a closed structure with two hydrogen bonds. The nucleobase and water can act as both the proton donor and acceptor. However, the obtained map also indicates that there is an area of positive charge between bond N(7)-H and C-H, where the hydrogen atom of water will be excluded. In this case 2TX can only be a proton donor. These results also indicate that apart from the area involved H-bond formation, the electrostatic potential of 2TX change little after formation of complexes.

**Figure 2:**
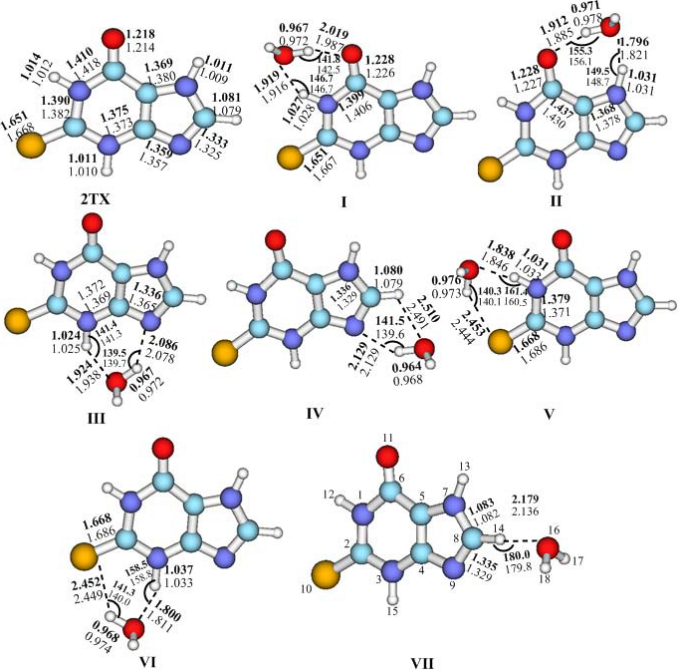
**Optimized structures of 2TX and its seven 2TX^...^H_2_O complexes at the MP2/6-311G(d, p) (bold) and B3LYP/6-311G(d, p) levels**. Bond distances and angles are in angstroms and degrees, respectively.

The MEP map provides us a clear indication of how water could interact with 2TX, in all seven 2TX^...^H_2_O complexes were identified, which supplies the initial structure for optimization. At the MP2 and B3LYP levels, these complexes have all real vibrational frequencies and represent minima on the potential energy surface. The optimized structures including geometry parameters and atomic number of the seven hydrated complexes are shown in Figure 2. Interaction energies, geometric and vibrational characteristics of the H-bonded complexes are listed in Table 1.

**Table 1:** Interaction energies including BSSE corrections (Δ*E*) of the seven 2TX^...^H_2_O complexes, BSSE, bond lengths of X--H (*R*_X--H_), hydrogen bond distances (*r*_Y...H_), selected change of X--H bond lengths (Δ*r*_X-H_), stretching vibrational frequency of monomer upon complex formation (Δ*ν*_X-H_), and the corresponding calculated change of infrared intensities (Δ*I*_X-H_) at the MP2/6-311G (d, p) level.

complexes	**Δ*E***^**a**^	**BSSE**^**a**^	X--H^...^Y	***R***_**X-H**_^**b**^	***r***_**Y^...^H**_^b^	**Δ*r***_**X-H**_^**b**^	**Δ*ν***_**X-H**_^**c**^	**Δ*I***_**X-H**_^**d**^
**I**	-7.6^e^	1.6^e^	N(1)--H^...^O_w_^g^	1.027	1.919	+0.013	--230	+436
	(--6.6)^f^	(0.6)^f^	O_w_--H^...^O(11)^h^	0.967	2.019	+0.009	--112	+134
**II**	--12.7	1.9	N(7)--H^...^O_w_	1.031	1.796	+0.020	--344	+642
	(--11.7)	(0.6)	O_w_--H^...^O	0.971	1.912	+0.013	--194	+402
**III**	--8.6	1.6	N(3)--H^...^O_w_	1.024	1.924	+0.013	--216	+386
	(--6.8)	(0.6)	O_w_--H^...^N	0.967	2.086	+0.009	--124	+158
**IV**	--4.8	1.0	C--H^...^O_w_	1.080	2.510	-0.001	+10	+19
	(--3.6)	(0.4)	O_w_--H^...^N	0.964	2.129	+0.005	--73	+145
**V**	--8.2	1.8	N(1)--H^...^O_w_	1.031	1.838	+0.017	--313	+504
	(--7.0)	(0.6)	O_w_--H^...^S	0.976	2.453	+0.018	--131	+152
**VI**	--9.3	1.8	N(3)--H^...^O_w_	1.037	1.800	+0.016	--366	+591
	(--8.2)	(0.6)	O_w_--H^...^S	0.968	2.452	+0.010	--225	+436

**VII**	--3.3	1.8	C--H^...^O_w_	1.083	2.179	+0.002	--31	+133
	(--2.6)	(0.4)						

^a ^kcal mol^-1^;^b ^Å;^c^cm^-1^; ^d ^km mol^-1^; ^e^calculated at the MP2/6-311++G(2d, 2p)//MP2/6-311G(d, p) level; ^f^calculated at the B3LYP/6-311++G(2d,2p)//B3LYP/6-311G(d, p) level; ^g^O_w _represents the oxygen atom of water.^h^change in the O--H symmetrical stretch frequencies of water, the change in asymmetric stretch is small and not given here.

As shown in Figure 2 and Table 1, the MP2 calculated bond lengths, angles, and interaction energies are very close to the B3LYP estimations. Unless specifically stated, those computed values discussed in the following sections are obtained from the MP2 method.

From Figure 2 it can be seen that only complex **VII **has an "open structure", whereas the other six hydrated 2TX complexes are "closed" with two nonlinear H-bonds [12]. Five different types of H-bond, denoted N-H^...^O, O-H^...^N, O-H^...^O, O-H^...^S and C-H^...^O can be found in these complexes, listed in Table 1. Analysis of the complexes' hydrogen bond distances revealed that the C-H^...^O distance in complex **IV **(2.510 Å) is considerably longer than those of the other four types of H-bond, which indicates this H-bond is the weakest one. The N-H^...^O distances in complexes **I**, **II**, **III**, **VI **and **V **are in the region of 1.79-1.92 Å while the O-H^...^N H-bonds in **III **and **IV **are longer, 2.08-2.12 Å. The O-H^...^O distance in structures **I**, **II**, **III **and **IV **are in the range of 1.91 to 2.01 Å. The sulfur atom in 2TX can also function as a proton acceptor and interact with water, but the O-H^...^S distance is relatively long at approximately 2.45 Å.

Compared with the isolated 2TX parent monomer, the core structure of 2TX in the identified complexes is little changed although some of the bonds involved in the formation of H-bonds are modified. All the N-H bonds interacting with H_2_O are elongated by about 0.0138-0.020 Å, the O-H bond of water in the complexes is also longer than that of in an isolated H_2_O molecule. These elongations of the proton donor X-H bond display the characteristic classical red-shifting H-bonds, it is noteworthy that the C-H bond in complex **IV **is shortened by about 0.001 Å, which indicates that the C-H^...^O may be an improper blue-shifting H-bond.

As shown in Table 1, the interaction energies (Δ*E*) with BSSE corrections are all negative, which indicates that the hydrogen bonds do indeed stabilise the complexes. The MP2 method shows Δ*E *to be very close to that obtained from B3LYP, but the B3LYP BSSE is a little smaller than that obtained by MP2. At the MP2 level of theory, the calculated Δ*E *of the seven H-bond complexes is about 3-13 kcal mol^-1^. Generally, based on the interaction energy, the H-bonds can be classified into strong (15-45 kcal mol^-1^), medium (4-15 kcal mol^-1^), and weak (1-4 kcal mol^-1^) [63]. The present results show that medium H-bonds are formed in the most hydrated 2TX complexes. Complex **VII**, however, has a weak H-bond with a Δ*E *of -3.3 kcal mol^-1^, and is the least stable structure of the seven 2TX^...^H_2_O complexes. It is well known that long range dispersion interactions play a dominant role in weak intermolecular interactions [64]. Whilst MP2 theory considers dispersion energy the B3LYP method does not account for such long-range correlations [64]. The weak H-bond of the complex **VII**, the B3LYP Δ*E *is -2.6 kcal mol^-1^, which is very close to the MP2 result (-3.3 kcal mol^-1^). This result indicates that dispersion forces make little contribution to the interaction between water and 2TX, as such our results indicate that the B3LYP method is also able to provide reliable results. This may be useful when larger systems prohibitive to the MP2 estimation are studied. As such B3LYP calculations could be used for the study of H-bonds in such a system.

Among the seven hydrated complexes, the structure **II **is the most stable. At the MP2/aug-cc-pVDZ//MP2/6-311G(d, p) and B3LYP/6-311++G(d, p)//B3LYP/6-311G(d, p) level, its interaction energies are -12.1 and -12.7 kcal mol^-1^, respectively. The values are larger than those of other hydrated nuclear bases such as uracil (-9.6 kcal mol^-1 ^at the MP2/aug-cc-pVDZ level) [15], cytosine (-8.6 kcal mol^-1 ^at the B3LYP/6-311++G(d, p) level) [17] and thymine (-7.9 kcal mol^-1 ^at the B3LYP/6-311++G(d, p) level)[21]. The results indicate that the H-bond in the hydrated 2TX complex is a little stronger than those in the other hydrated nuclear base complexes.

Structure **II **has the shortest N-H^...^O distance of 1.796 Å. The N-H^...^O hydrogen distance of the complex **VI **is 1.800 Å, but the O-H^...^S hydrogen bond in this system is weaker than the O-H^...^O hydrogen bond of complex **II**. Thus, the Δ*E *of the former is 4.5 kcal mol^-1 ^smaller than that of the latter. Complex **IV **has two H-bonds but it is the second least stable structure among the hydrated complexes studied. Its Δ*E *is just 1.5 kcal mol^-1 ^larger than that of complex **VII **(the least stable). The hydrogen bond angle ∠C-H^...^O is 109.5° for complex **IV**, which is much smaller than those of other complexes. The bent structure of **IV **results in a weak H-bond with a small interaction energy.

Why then do the seven 2TX-water complexes have different interaction energies? For instance, the structures **I **and **II **have similar closed O^...^HO^...^HN hydrogen bonds but markedly different interaction energy of -7.6 and -12.7 kcal mol^-1^, respectively. The difference may be due to their different acidities of the N(1)-H and N(7)-H. To address these questions, we calculated proton affinities (PEs) and deprotonantion enthalpies (DPEs) [17] of the sites involved in the H-bond formation of 2TX. The computed PA of O, N, and S atoms and DEP of N-H and C-H bond are collected in Table 2.

**Table 2:** MP2/6-311++G(2d,2p)//MP2/6-311G(d, p) proton affinity (PA) of O, S, and N atoms, deprotonation enthalpy (DPE) of N--H and C--H bonds in the 2TX, and 1.5DPE--PA of seven 2TX^...^H_2_O complexes. All the values are in kcal mol^-1^.

atoms and bonds	PA	DPE	complexes	1.5DPE--PA
O(11) (N(1) side)	198.2		**I**	303.0
O(11) (N(7) side)	197.0		**II**	280.0
S(10) (N(1) side)	205.8		**III**	287.2
S(10) (N(3) side)	205.6		**IV**	335.7
N(9)	203.9		**V**	295.4
N(1)--H		334.1	**VI**	285.5
N(3)--H		327.4	**VII**	539.6
N(7)--H		318.0		
C(8)--H		359.7		

Zeegers-Huyskens and co-workers have reported theoretical studies on acidity and basicity of guanine, adenine, urail, thymine, and cytosine[14,16-19,23]. Their results suggest that hydrogen bond energies of these hydrated nucleobases are correlated to the PA and DPE of the sites involved in interaction with water. They obtained a good quantitative relationship between the Δ*E *and the values of 1.5DPE-PA [16,17,23], the smaller 1.5DPE-PA, the larger Δ*E*.

As shown in the Table 2, N(7)-H has the lowest DPE, which indicates that it has the strongest acidity and can form stronger hydrogen bonds. Among the O, N and S atoms, S11 has the highest PA of about 205 kcal mol^-1^, which indicates that it's basicity is strongest. Next, the values of 1.5DPE-PA for the complexes were calculated. Comparing Table 1 and Table 2, it can be seen that the complex with lower 1.5DPE-PA has larger Δ*E*. The order of 1.5DPE-PA for the seven complexes is consistent with that of the interaction energies. A good correlation between the interaction energy and the values of 1.5DPE-PA can be expressed by the following exponential expression:

Complexes **I **and **II **have close PAs but the latter's DPE is l6.1 kcal mol^-1 ^lower than that of the former. The much stronger acidity of N(7)-H compensates the lower basicity of O11 (N(7) site) in structure **II**. As a result, complex **II **has lower values of 1.5DPE-PA than **I**. In addition, the hydrogen bond angle ∠O-H^...^O in complexes is small compared to ordinary O-H^...^O H-bond angles, about 141.8°. The weaker acidity of N(1)-H and small hydrogen bond angle in complex **I **result in it being 5.1 kcal mol^-1 ^less stable than complexes **II**. Compared with structure **I**, complexes **III **and **VI **have lower DPEs of N(3)-H and higher PAs of the S atom. The stronger acidity and basicity of the sites involved the H-bond formation result in the two complexes are more stable than the complex **I**. The acidity of C(8)-H bond is the weakest, thus complex **VII **has the highest 1.5DPE-PA and is the least stable among the seven complexes. The relative stability order of the studied complexes can be explained by the acidity and basicity of the sites satisfactorily forming H-bonds, which agrees with previous conclusion on the cyclic hydrated nucleobases by Zeegers-Huyskens and coworkers [14,16-19,23].

In summary, the present studies on the hydration of 2TX show that the water can interact with the nucleobase in different sites to form various H-bonds. The intrinsic acidity and basicity of the sites forming H-bonds leads to the different interaction energies. When H_2_O approaches the N(7)-H site, a closed complex **II **with two H-bonds is the most stable. In the C-H site, complex **VII **with only one H-bond is the least stable one.

### Infrared and NMR spectroscopy

**Figure 3:**
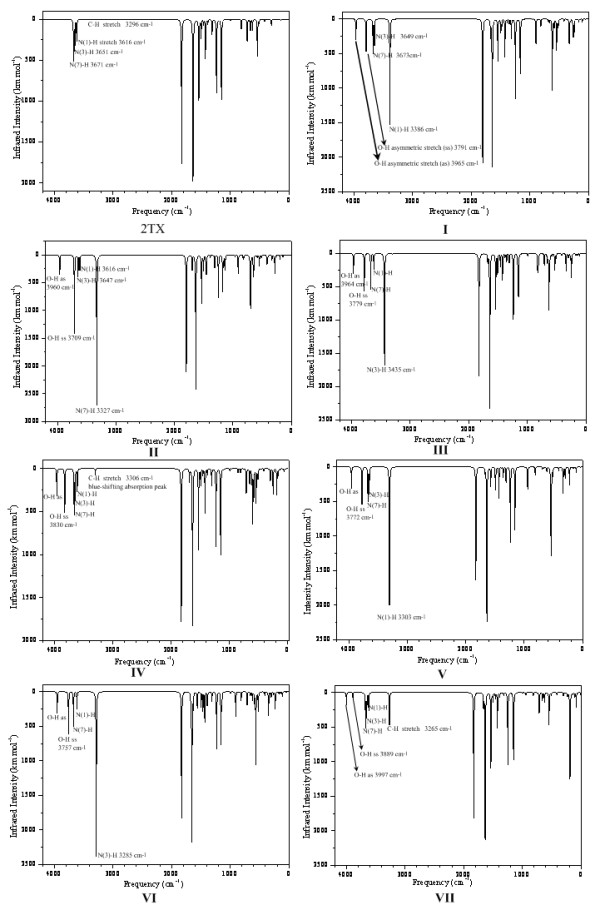
**Theoretical infrared spectrum of 2TX and its hydrated complexes at the MP2/6-311G(d, p) level**.

Infrared (IR) spectroscopy is a frequently used, useful spectroscopic tool to identify the existence of H-bonds [65]. NMR spectroscopy has become the second spectroscopic probe of choice for H-bonds which provides indirect evidence for the formation of H-bonds [66]. Here, the theoretical infrared and NMR spectra of hydrated 2TX complexes are reported to probe the characteristics of the proposed H-bonds. The calculated IR spectra are displayed in Figure 3. The computed ^1^H NMR chemical shifts in H-bonded complexes are listed in Table 2.

Comparing the IR spectra of the parent 2TX and hydrated 2TX, it can be seen that the latter is quite different, especially in the area from 3000 to 4000 cm^-1^. As show in the Figure 3, three weak peaks corresponding to N-H vibrational stretches and one very weak C-H stretch are observed, 3616 (N(1)-H), 3651 (N(3)-H), 3671 (N(7)-H), and 3296 cm^-1 ^(C-H). After forming H-bonds, a much stronger red-shifting band will appear in the area between 3000 and 4000 cm^-1 ^for the hydrated 2TX complexes. In Table 1, the changes of X-H bond lengths (Δ*r*_X-H_) and corresponding stretching vibrational frequencies upon complex formation (Δ*ν*_X-H_) are listed. The results indicate that most complexes have an elongated X-H bond with a red-shift stretching frequency of 78-366 cm^-1^. The relative infrared intensities also increase (100-600 km/mol). That these shifts can be observed in the theoretical IR spectra of the complexes, confirms the existence of the formation of H-bonds.

For complexes **I**, **II**, **III**, **V**, and **VI**, the N-H and O-H stretching frequency changes with a large red-shift upon H-bond formation, whereas in **IV **and **VII **the O-H and C-H stretching frequencies are a little altered. These results indicate that the N-H^...^O, O-H^...^O and O-H^...^S interactions are classical red-shifting H-bonds. However, the calculated IR spectrum of complex **IV **is distinctly different from those of the other complexes with two H-bonds. The complex has a slightly shortened C-H bond with a corresponding frequency of 3306 cm^-1^. Thus in contrast to the C-H stretching frequency of 3296 cm^-1 ^in 2TX, the C-H stretching frequency of complex **IV **has a blue-shift of 10 cm^-1^. This result indicates that the C-H^...^O H-bond in complex **IV **is not a classical red-shifting H-bond but an improper blue-shifting H-bond [67-69]. Complex **VII **also has a C-H^...^O bond, the relative C-H bond is elongated and the corresponding stretching frequency has a red-shift of 31 cm^-1^, i.e. a classical red-shifting H-bond.

2TX has four hydrogen atoms, N(1)H, N(3)H, N(7)H, and C(8)H (see scheme 1). Their calculated ^1^H NMR chemical shifts (*δ*) are 7.76, 8.75, 7.93 and 8.75 ppm, respectively. Twanmoh [41]reported the experimental NMR spectrum of 2TX in DMSO solution. The oxygen atom of DMSO can interact with the hydrogen atoms of 2TX. Thus, the corresponding proton chemical shifts of 2TX increase. Kupka [70]and Hannongbua [71]reported the experimental NMR spectra of uracil and nevirapine in DMSO, respectively. Their results show that the hydrogen bonds between the acidic protons and polar solvent molecules leads to the augmentation of the corresponding proton chemical shifts. As shown in Table 2, after the formation of H-bonds in complexes, the computed ^1^H NMR chemical shifts of H-bonded protons increases, whereas the non-H-bonding protons change very little. The proton of N(7)H, complex **II**, has a chemical shift of 13.2 ppm, whilst in the parent structure it is 8.75 ppm. The calculated chemical shift of 13.2 ppm for N(7)H is very close to the experimental values of 13.3 ppm in DMSO solution [41]. The theoretical results show that the increase in *δ *of the H-bonded N-H proton in the complex is about 3-5 ppm, whilst for the H-bonded C-H proton, the changes are about 0.5-1.5 ppm. In complexes **IV **and **VII**, the C-H^...^O bond are improper blue-shifting and classical red-shifting H-bonds respectively, both of these types of H-bond give an increased ^1^H NMR chemical shift, with the largest increases being due to the classical red-shifting H-bond.

**Figure 4:**
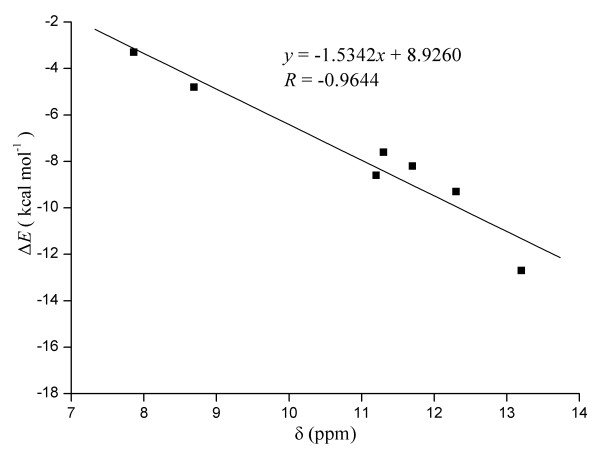
**Plot of MP2/6-311G(d, p) interaction energy (Δ*E*) versus MP2/6-311G(d, p) NMR proton chemical shifts (*δ*) of the H-bonded complexes**.

Del Bene *et al*. [72]studied a series of H-bonded complexes with MP2 theory. They found a correlation between the increasing chemical shift for the hydrogen bonded protons and binding energy. It can be seen in Table 1 and 2, the larger interaction energy of complexes, the greater the corresponding proton NMR chemical shift increases. The most stable complex (**II**) has the highest ^1^H NMR chemical shift of 13.2 ppm. Figure 4 presents a plot of the calculated interaction energy versus relative H-bonded proton NMR chemical shift. A good linear correlation (correlation coefficient 0.96) between computed Δ*E *and chemical shifts was obtained.

These results indicate that after formation of H-bonds, IR and NMR spectra change greatly, which further facilitates the identification of hydration of 2TX.

### NBO and AIM analysis

For better understanding of the bonding characteristics in the hydrated 2TX complexes, NBO and AIM analysis were carried out at the MP2/6-311G(d, p) level.

The calculated NBO charges show that forming an H-bond will result in hydrogen possessing a greater positive charge, whereas X and Y have a more negative charge. But for the complex **IV**, the C atom of the C-H^...^O hydrogen bond has more positive charge. Electron density transfer (EDT) from water to 2TX for the most complexes is positive but the charge is transferred from 2TX to water in the complex **IV**. Thus, the improper blue-shifting C-H^...^O H-bond has different bonding characteristics from the other classical H-bonds.

According to NBO analysis, the second-order perturbation stabilization energy Δ*E*(2) between the lone pair (LP) orbital of atom Y and σ antibonding orbital (σ*) of X-H bond are computed and presented in the Table 3. Δ*E*(2) is calculated by second-order perturbation theory analysis of the Fock matrix [49] and associated with a charge transfer interaction between the relevant donor-acceptor orbitals.

**Table 3:** GIAO MP2/6-311G(d, p) calculated ^1^H chemical shielding (*δ*/ppm) for isolated and monohydrated 2TX

atom number	**H**_**2**_**O**	2TX	I	II	III	IV	V	VI	VII
12	---	7.76	**11.25**	7.87	7.74	7.76	**11.67**	7.84	7.72
13	---	8.75	8.72	**13.15**	8.75	8.78	8.76	8.73	8.78
14	---	7.24	7.23	7.27	7.16	**7.86**	7.22	7.26	**8.69**
15	---	7.93	7.96	8.07	**11.21**	7.83	8.01	**12.33**	7.88
17	0.11	---	2.87	4.68	3.49	2.74	3.13	3.32	0.70
18	0.11	---	1.11	1.17	1.16	0.19	1.21	1.37	0.70

**Figure 5:**
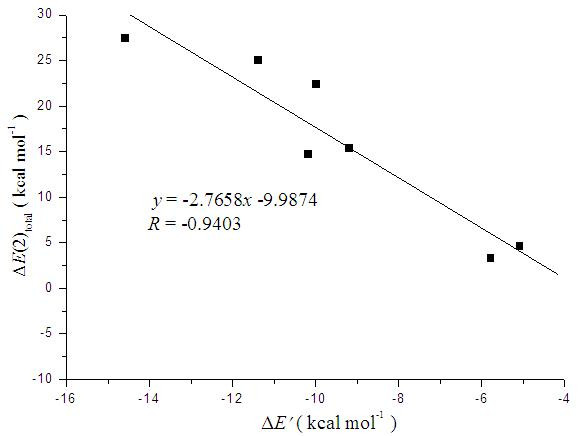
**Plot of total hydroconjugation energies (Δ*E*(2)_total_) versus interaction energy without BSSE correction (Δ*E*') of the H-bonded complexes**.

Previous studies [73]of H-bonded systems containing N-H^...^X bonds show that the Δ*E*(2) has a excellent linear correlation with the interaction energy, correlation coefficient 0.99. As shown in Figure 5, a linear correlation between the interaction energy without BSSE correction (Δ*E'*) and total hydroconjugation energies (Δ*E*(2)_total_) was also found, which has a better correlation coefficient than considering the interaction energy including BSSE corrections (*r *= 0.9155). The Δ*E*(2) between the LP of atom Y and σ* of the X-H bond correlates to the intensity of the X-H^...^Y hydrogen bond. The Δ*E*(2) of the X-H^...^Y interaction can provide a qualitative description of the contribution to the total interaction energy of the complexes. For example, in complexes such as **II**, the Δ*E*(2) of N-H^...^O are larger than that of the O-H^...^O bond, which indicates the N-H^...^O H-bond makes more of a contribution towards the interaction energy of the complexes. These qualitative conclusions agree with the fact that the N-H^...^O H-bond is shorter than the O-H^...^O bond.

**Figure 6:**
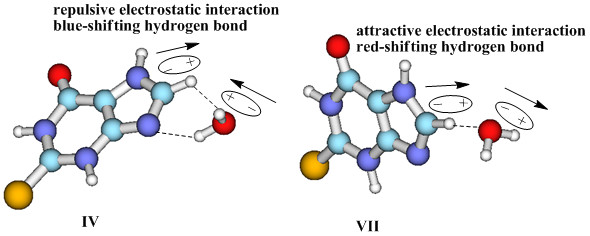
**Different dipole orientations of water in complexes IV and VII**.

Comparison of complexes **IV **and **VII**, reveals both hydrated complexes to have C-H^...^O H-bonds and a small Δ*E*. In the former, the C-H^...^O interaction is an improper blue-shifting H-bond, whereas the latter has a classical C-H^...^O H-bond. Why then do different types of H-bond form in the two complexes? The NBO analysis shows that the Δ*E*(2) of C-H^...^O is almost zero (see Table 4). To explore the origin of blue-shifting H-bonds, Li and Schlegel [68] studied many blue-shifting H-bonded systems and found that the absence of orbital interactions cause bond elongation, the repulsive interaction between two fragments results in the compression of X-H and a relative blue-shift of the vibration frequency. Analyzing the orbital interaction and electrostatic interaction of complexes **IV **and **VII**, it was found that the orbital interactions of C-H^...^O in **IV **are very weak (the related Δ*E*(2) is almost zero) and the electrostatic interaction between the H_2_O molecule and N-H moiety of 2TX is repulsive. But in complex **VII **medium orbital interactions (the corresponding Δ*E*(2) is 4.7 kcal mol^-1^), which lead to the elongation of C-H and a red shift are present. Figure 6 shows the different dipole orientation of the water in complexes **IV **and **VII**. The orientation was drawn qualitatively according to the molecular electrostatic potential of the two complexes in Figure 1. The different dipole orientation of the water leads to the former having a repulsive electrostatic interaction, as opposed to the latter having an attractive one. The different orbital interactions and electrostatic forces of the two complexes result in the different types of H-bond.

**Table 4:** Second-order perturbation stabilization energies Δ*E*(2) (kcal mol^-1^) of the H-bonded complexes

complexes	donor NBOs	acceptor NBOs	Δ*E*(2)
**I**	LP(O11)^a^	σ* (H17-O16)	4.2
	LP(O16)	σ* (N1-H12)	11.2
**II**	LP(O11)	σ* (H17-O16)	9.7
	LP(O16)	σ* (N7-H13)	17.8
**III**	LP(N3)	σ* (H17-O16)	4.5
	LP(O16)	σ* (N3-H15)	10.4
**IV**	LP(N11)	σ* (H17-O16)	3.0
	LP(O16)	σ* (C8-H14)	0.4
**V**	LP(S10)	σ* (O16-H17)	6.3
	LP(O16)	σ* (N1-H12)	16.3
**VI**	LP(S10)	σ* (O16-H17)	6.6
	LP(O16)	σ* (N3-H15)	18.5
**VII**	LP(O16)	σ* (C8-H14)	4.7

^a^LP = lone pair orbital; ^b ^σ * = σ antibonding orbital.

AIM theory has been successfully applied to characterizing hydrogen bonds of a series of H-bonded complexes [58,59]. Popelier [58]proposed a set of criteria for the existence of H-bonding based on AIM analysis. These criteria for hydrogen bonding are: (1) Correct topological pattern (i.e., the existence of a bond critical point (BCP) and a bond path); (2) proper value of electron density; (3) the Laplacian of electron density at the BCP; (4) mutual infiltration of H and Y atoms; (5) loss of hydrogen atom net charge; (6) energetic destabilization of hydrogen; (7) decrease of dipolar polarization; (8) decrease of hydrogen atomic volume. Here, these eight criteria are used to examine the studied H-bonded complexes.

**Figure 7:**
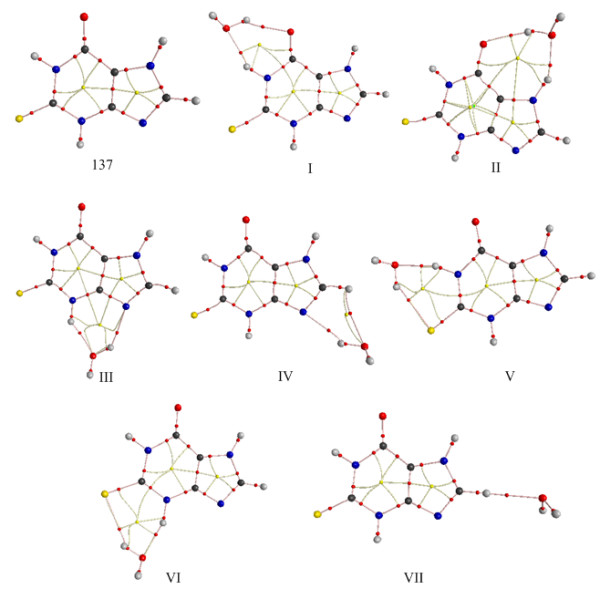
**Molecular graphs of the seven H-bonded complexes by AIM theory analysis. Small red and yellow dots represent the bond critical point and ring critical point, respectively**.

Figure 7 demonstrates the existence of two BCPs and the relative bond paths of six complexes with two H-bonds. For complex **VII**, one BCP and bond path can be found. For the six closed H-bonded systems, a ring critical point (RCP) is present in the centre of the ring. But for complex **IV**, the RCP is very close to the BCP between the C-H bond and the O atom of water.

Table 4 lists the topological properties of the BCP of the H-bonded complexes. The electron density (*ρ*) at the BCP varies from 0.0156 to 0.0335 au, which falls within the proposed range of 0.002-0.035 au for H-bonds. It has been shown the *ρ *at the BCP correlates with the interaction energy and H-bond length [58]. As shown in Table 4, the *ρ *of the N-H^...^O bond is larger than that of the O-H^...^O bond, which is consistent with the N-H^...^O interaction having a shorter H-bond distance.

The third necessary criterion focuses on the Laplacian of the charge density (∇^2^*ρ*) at the BCP. It has been observed that for closed-shell interactions including ionic bonds, hydrogen bonds and van der Waals interactions, the ∇^2^*ρ *is positive [58]. It can be seen that all the ∇^2^*ρ *at the BCP of the present studied H-bonds are positive ~0.0446-0.1009 au, which lies within the proposed range of 0.024 to 0.139 au [58,59].

In order to estimate the mutual penetration of hydrogen (H) and acceptor atom (Y) upon H-bond formation, the nonbonded radius of these atoms ( And ) have to be compared to the relative bonded radius (*r*_H _and *r*_Y_). The nonbonded radius is defined as the distance of its nucleus to an electron density of 0.001 au [58]. The bonded radius is the distance between the nucleus and the corresponding BCP. The penetration is defined as the nonbonded radius minus bonded radius [58]. According to the Table 5, in all O-H^...^O H-bonds and most N-H^...^O H-bonds, the heteroatom is penetrated more than the H atom. But in the C-H^...^O bond, the hydrogen atom is penetrated more than the oxygen, which is consistent with a system reported by Popelier [58]. Thus the data shows a mutual penetration between the hydrogen and heteroatom H-bond acceptor.

**Table 5:** Electron density topological properties at the BCP of the X--H^...^Y H-bonds in the 2TX^...^H_2_O complexes.

complexes	interactions	*ρ*	**∇**^**2**^***ρ***	*ε*	**Δ*r***_**H**_	**Δ*r***_**Y**_	**Δ*r***_**H**_**+Δ*r***_**Y**_
**I**	N(1)--H^...^O	0.0269	0.1009	0.0495	0.9885	1.0125	2.001
	O--H^...^O	0.0206	0.0840	0.0205	0.702	0.8135	1.5155
**II**	N(7)--H^...^O	0.0316	0.0877	0.0920	1.0914	1.0952	2.1866
	O--H^...^O	0.0224	0.0741	0.1277	0.3525	0.9298	1.2823
**III**	N(3)--H^...^O	0.0178	0.0669	0.0860	0.9108	1.0213	1.9321
	O--H^...^O	0.0129	0.0446	0.1155	0.6701	1.0385	1.7086
**IV**	C--H^...^O	0.0101	0.0405	2.1209	0.4934	0.4889	0.9823
	O--H^...^O	0.0218	0.0764	0.0304	0.657	0.9505	1.6075
**V**	N(1)--H^...^O	0.0306	0.1198	0.0308	1.0878	1.0732	2.161
	O--H^...^S	0.0170	0.0489	0.0333	0.6224	0.6248	1.2472
**VI**	N(3)--H^...^O	0.0335	0.1292	0.0308	1.0782	1.109	2.1872
	O--H^...^S	0.0171	0.0486	0.0348	0.6271	0.5859	1.213
**VII**	C--H^...^O	0.0156	0.0585	0.0317	0.9145	0.7611	1.6756

Electron Density (*ρ*), Laplacian (∇^2^*ρ*), Ellipticity (*ε*), the difference of atomic radius (Δ*r*) between monomer and H-bonded complexes. All units are atomic units.

The integrated atomic properties for the hydrogen atoms of the X-H^...^Y H-bond and the difference of the properties between the complexes and parent molecule are collected in Table 6. All the changes show the same expected trends as for the H-bonding system by Popelier [58].

**Table 6:** Atomic basin integrated properties^a^of the hydrogen atoms in H-bond interactions and the relative difference between H-bonded complexes and parent molecule.

complexes	interaction	*q*	Δ*q*	*E*	Δ*E*	|*M*|	Δ|*M*|	*ν*	Δ*ν*
**I**	N(1)--H^...^O	0.449	-0.075	-0.373	0.041	0.110	-0.043	17.43	-9.52
	O--H^...^O	0.346	-0.156	-0.32	0.101	0.125	-0.037	14.83	-10.05
**II**	N(7)--H^...^O	0.293	-0.223	-0.274	0.14	0.088	-0.059	14.93	-10.93
	O--H^...^O	0.253	-0.249	-0.265	0.156	0.101	-0.061	14.41	-10.47
**III**	N(3)--H^...^O	0.213	-0.301	-0.235	0.178	0.082	-0.068	13.61	-12.55
	O--H^...^O	0.170	-0.332	-0.235	0.186	0.080	-0.082	12.79	-12.09
**IV**	C--H^...^O	0.751	-0.153	-0.512	0.071	0.104	-0.006	37.46	-8.08
	O--H^...^O	0.371	-0.131	-0.33	0.091	0.14	-0.022	16.28	-8.60
**V**	N(1)--H^...^O	0.44	-0.084	-0.37	0.044	0.108	-0.045	15.00	-11.95
	O--H^...^S	0.369	-0.133	-0.33	0.091	0.145	-0.017	16.54	-8.34
**VI**	N(3)--H^...^O	0.425	-0.089	-0.361	0.052	0.105	-0.045	14.23	-11.93
	O--H^...^S	0.365	-0.137	-0.327	0.094	0.144	-0.018	16.28	-8.60
**VII**	C--H^...^O	0.828	-0.076	-0.549	0.034	0.085	-0.025	36.52	-9.02

^a^All values in atomic units: *q*, net charge; *E*, atomic energy; |*M*|, first moment; *ν*, volume.

From the preceding discussion, the eight AIM criteria of hydrogen bonds are all echoed in the studied H-bonding interactions in the hydrated 2TX complexes. Electron density topological analysis does not show any obvious difference between the classical red-shifting and improper blue-shifting H-bonds. The classical red-shifting and improper blue-shifting H-bonds have consistent changes including the loss of charge, energetic destabilization, decrease of dipolar polarization, and decreased volume of the hydrogen atom in H-bonds.

## Conclusion

MP2 and B3LYP calculations have been carried out to study the interactions between of 2-thioxanthine and water 1:1 complexes. Seven theoretical monohydrated complexes have been identified and reported for the first time. Harmonic vibrational frequency analysis confirms that these complexes are minima on the potential energy surface. The MP2 calculated bond lengths, angles and interactions energy are very close to the B3LYP results, which indicates the B3LYP method could also be a suitable tool for the study of similar H-bonded systems. Among the seven hydrated complexes, the closed complex **II **with N(7)-H^...^O and O-H^...^O hydrogen bonds is the most stable. The hydrogen bond energies of the complexes are correlated to the PA and DPE of the sites involved in interaction with water. The theoretical IR spectra show that most complexes have classical H-bonds with a stretching frequency red-shifted by 78-366 cm^-1 ^accompanied by an increase in the relative infrared intensities of 100-600 km mol^-1^. An improper blue-shifting H-bond with a shortened C-H bond was found in complex **IV**. The theoretical GIAO NMR estimations show that the H-bonded proton of the complex has an increased chemical shift. There is a good linear correlation between the Δ*E *and *δ *of the H-bonded proton. NBO analysis shows that the absence of orbital interactions and repulsive electrostatic interactions results in the formation of a blue-shifting H-bond in complex **IV**. The eight AIM criteria of hydrogen bonds suggested by Popelier are all met in the studied H-bonding systems. However, electron density topological analysis does not show an obvious difference between the classical red-shifting and improper blue-shifting H-bonds.

## Competing interests

The authors declare that they have no competing interests.

## Authors' contributions

XXY carried out the MP2, DFT and AIM calculations and wrote some portions of the paper. YFW preformed the NBO analysis. XW and JSF conceived of the study, collected and analysed all the data, discussed the results and wrote the manuscript. WC analysed the IR and NMR spectra. NBW discussed the results and revised the manuscript.
